# Potential of Maintaining a Healthy Vaginal Environment by Two* Lactobacillus* Strains Isolated from Cocoa Fermentation

**DOI:** 10.1155/2018/7571954

**Published:** 2018-09-30

**Authors:** Ana Clara Correia Melgaço, Wallace Felipe Blohem Pessoa, Herbert Pina Freire, Milena Evangelista de Almeida, Maysa Santos Barbosa, Rachel Passos Rezende, Jorge Timenetsky, Lucas Miranda Marques, Carla Cristina Romano

**Affiliations:** ^1^Departamento de Ciências Biológicas, Laboratório de Imunologia, Centro de Biotecnologia e Genética, Universidade Estadual de Santa Cruz (UESC), Campus Soane Nazaré de Andrade, Salobrinho, Rodovia Jorge Amado, Km 16, 45662-900 Ilhéus, BA, Brazil; ^2^Instituto de Ciências Biomédicas, Departamento de Microbiologia, Laboratório de Micoplasmas, Universidade de São Paulo (USP), São Paulo, Brazil; ^3^Departamento de Ciências Biológicas, Laboratório de Biotecnologia Microbiana, Centro de Biotecnologia e Genética, Universidade Estadual de Santa Cruz (UESC), Campus Soane Nazaré de Andrade, Salobrinho, Rodovia Jorge Amado, Km 16, 45662-900 Ilhéus, BA, Brazil; ^4^Instituto Multidisciplinar em Saúde/Campus Anísio Teixeira, Universidade Federal da Bahia, IMS/CAT-UFBA, Vitória da Conquista, Brazil

## Abstract

Bacteria in the genera* Mycoplasma* and* Ureaplasma* do not have cell walls and therefore interact with host cells through lipid-associated membrane proteins (LAMP). These lipoproteins are important for both surface adhesion and modulation of host immune responses.* Mycoplasma* and* Ureaplasma* have been implicated in cases of bacterial vaginosis (BV), which can cause infertility, abortion, and premature delivery. In contrast, bacteria of the genus* Lactobacillus*, which are present in the vaginal microbiota of healthy women, are thought to inhibit local colonization by pathogenic microorganisms. The aim of the present study was to evaluate the* in vitro* interactions between lipoproteins of* Mycoplasma* and* Ureaplasma* species and vaginal lineage (HMVII) cells and to study the effect of* Lactobacillus* isolates from cocoa fermentation on these interactions. The tested* Lactobacillus* strains showed some important probiotic characteristics, with autoaggregation percentages of 28.55% and 31.82% for* L. fermentum* FA4 and* L. plantarum* PA3 strains, respectively, and percent adhesion values of 31.66 and 41.65%, respectively. The two strains were hydrophobic, with moderate to high hydrophobicity values, 65.33% and 71.12% for* L. fermentum* FA4 and* L. plantarum* PA3 in toluene. Both strains secreted acids into the culture medium with pH=4.32 and pH=4.33, respectively, and showed antibiotics susceptibility profiles similar to those of other lactobacilli. The strains were also able to inhibit the death of vaginal epithelial cells after incubation with* U. parvum* LAMP from 41.03% to 2.43% (*L. fermentum* FA4) and 0.43% (*L. plantarum* PA3) and also managed to significantly decrease the rate of cell death caused by the interaction with LAMP of* M. hominis* from 34.29% to 14.06% (*L. fermentum* FA4) and 14.61% (*L. plantarum* PA3), thus demonstrating their potential for maintaining a healthy vaginal environment.

## 1. Introduction

Bacteria of the genera* Mycoplasma* and* Ureaplasma *belong to the class* Mollicutes*, and these microorganisms are the smallest known free-living organisms. With genomes of only 580–2,200 kb, depending on the species, these bacteria contain only the minimal structures necessary for cell growth and replication and are unable to synthesize some substances that are essential for their growth. Therefore, these substances must be obtained from their hosts [1, 2]. Because they do not have cell walls, these bacteria contact host cells through their plasma membrane, which is composed of a lipid-protein bilayer. Mycoplasmas lipid-associated membrane proteins (LAMP) play an important role in both adhesion to the host cell surface and immune response modulation through the production of proinflammatory cytokines. They also induce apoptosis in different types of cells, such as monocytes and macrophages [3–5]. Mycoplasmas and ureaplasmas are pathogens that are frequently associated with mucosal infections of the respiratory and urogenital tracts [1, 2]. In the female genital tract, mycoplasmas and ureaplasmas have been directly implicated in cases of bacterial vaginosis (BV) [6, 7].

BV is a syndrome resulting from an imbalance of the vaginal microbiota, with concomitant proliferation of pathogenic bacteria. These infections mainly affect women of childbearing age, and they have been associated with infertility, preterm birth, endometritis, pelvic inflammatory disease, and susceptibility to infection with human immunodeficiency virus (HIV) [6, 8, 9]. In addition to* Mycoplasma* and* Ureaplasma* species, the other major bacterial species related to BV belong to the genera* Chlamydia*,* Neisseria*, and* Gardnerella* [10]. Vaginal microbiota is considered to be healthy when specific bacterial community types that have beneficial functions for the host are present, along with the absence of clinical symptoms [11]. The vaginal microbiota of symptom-less women generally includes species of the genus* Lactobacillus*; these bacteria produce lactic acid, hydrogen peroxide (H_2_O_2_), bacteriocins, and hydroxyl radicals and thereby inhibit local colonization by pathogenic microorganisms. In addition, the presence of lactobacilli favors a protective environment for the fetus in pregnancy [10].

Lactobacilli as well as bacteria of the genus* Bifidobacterium, Lactococcus lactis*, and* Escherichia coli* as well as the yeast* Saccharomyces cerevisiae* have been used as probiotics, mainly by the food industry [12]. Probiotics are living microorganisms that, when administered in adequate quantities, confer benefits to host health [13]. Generally, lactobacilli in probiotic formulations were isolated from human microbiota; however, in recent years there has been growing interest in the use of strains isolated from nonhuman sources, including fermented foods, such as cocoa. Thus, in several studies, the probiotic potential of strains isolated from food fermentation has been investigated [14, 15]. Studies previously conducted by our research group showed that* Lactobacillus* strains derived from the fermentation of cocoa exhibited probiotic potential and antibiotics activity against distinct pathogens. Different strains reduced histological damage and the systemic concentration of inflammatory cytokines and elevated serum levels of immunoglobulin A (IgA) in a model of experimental colitis [16]. Culture supernatants of* L. fermentum* TCUESC01 and* L. plantarum *TCUESC02 inhibited growth and reduced the biofilm formation ability of streptomycin- and dihydrostreptomycin-resistant strains of* Staphylococcus aureus* [17]. They also showed antagonistic activity against* G. vaginalis* [18]. The aim of this study was to evaluate the* in vitro* interaction of lipoproteins from genital human* Mycoplasma* and* Ureaplasma* species with the HMVII vaginal cell line and to study the effect of* Lactobacillus *on this interaction.

## 2. Materials and Methods

### 2.1. Cell Lines, Microorganisms, and Growth Conditions

HMVII human vaginal epithelial cell line (BCRJ 0316) was obtained from the Rio de Janeiro Cell Bank and was grown in RPMI 1640 medium, supplemented with 10% fetal bovine serum (FSB), penicillin 100 IU/mL, and streptomycin 100 *μ*g/mL.

*L. fermentum* FA4 and* L. plantarum* PA3 were previously isolated by our research group from a cocoa fermentation [16]. These strains were confirmed to the species level by 16S rRNA sequencing and were deposited in GenBank (http://www.ncbi.nlm.nih.gov/) under accession numbers KU244506 and KU244472, respectively. Lactobacilli were grown in de Man, Rogosa, and Sharpe (MRS) medium for 18 h at 37°C in anaerobic jar.

*M. hominis *(ATCC 23114) and* M. genitalium *(ATCC 33530) were grown in 1 L of SP-4 medium.* U. urealyticum* serotype 7 (ATCC 27819) and* U. parvum* serotype 3 (ATCC 27815) were cultured in 200 mL of Ureaplasma Broth (UB) medium. All strains were maintained at 37°C and 5% CO_2_ until log phase growth. Growth control was observed by the observation of color change in the liquid medium, plus pH indicator (phenol red).

### 2.2. Extraction of Membrane-Associated Lipoproteins (LAMP)

Lipoproteins were extracted according to the method developed by Wang et al. [19] with some modifications. Briefly,* M. hominis*,* M. genitalium*,* U. parvum*, and* U. urealyticum* were cultured until log phase, until the observation of color change in the liquid medium, pH indicator (phenol red). Then, the cells were recovered by centrifugation at 23,700 ×*g *for 30 min at 4°C (Beckman Coulter) and washed with sterile phosphate-buffered saline (PBS) (1X, pH 7,4) to remove the residual culture medium. The cell pellet was suspended in 5 mL of Tris-EDTA (50 mM Tris [pH 8], 0.15 M NaCl, 1 mM EDTA), and Triton TX-114 was added to a final concentration of 2%. The mixture was homogenized by vortexing and incubated at 4°C for 60 min. The lysate was then incubated at 37°C for 10 min and centrifuged at 23,700 ×* g* at 22°C for 20 min for phase separation. The upper aqueous phase was discarded, and in the final TX-114 step, the volume was adjusted to the original volume by the addition of Tris-EDTA. Then, 2.5 volumes of ethanol were added to precipitate the lipoproteins overnight at −20°C. The precipitated materials were recovered by centrifugation at 23,700 ×*g *for 15 min at 4°C. After centrifugation, the pellet was homogenized in PBS by sonication, 6 to 8 cycles per minute at a power of 10W (Coler-Parmer Ultrasonic Processor). Then, the microtubes containing the lipoproteins were stored at −80°C until use. Lipoproteins were quantitated using the 2D Quant Kit (GE Healthcare) according to the manufacturer's protocol and preincubated for 2 h with polymyxin B at 1000 U/mL prior to the use.

### 2.3. Autoaggregation Assay

To verify the autoaggregation capacity of the* Lactobacillus* strains, the method of Kos et al. [20] was used, with some modifications. Briefly, the strains were cultured in MRS broth for 18 h at 37°C in anaerobic jar. Then, the cells were recovered by centrifugation (8000 ×* g*, 10 min), washed twice with 0.9% saline solution (w/v), and suspended in the same solution to 1 × 10^8^ CFU/mL in a spectrophotometer (Thermo-Scientific). The suspensions were homogenized by vortexing and incubated at 37°C for 5 h. Then, a 1 mL aliquot was gently removed from the top of the suspension every hour, and its absorbance at 600 nm (A_600_) was read in a spectrophotometer (Thermo-Scientific). Autoaggregation was calculated using the following formula: autoaggregation (%) = [(A_0_-A_t_)/A_0_] × 100, where A_0_ is the absorbance at time 0 h, and At is the absorbance at time t, which was measured every hour, for up to 5 h.

### 2.4. Hydrophobicity Assay

The hydrophobicity of the* Lactobacillus *strains was verified by testing microbial adherence to hydrocarbons (MATH), using a method adapted from Rodríguez et al. [21].* Lactobacillus* strains were cultured in MRS broth at 37°C for 18 h in anaerobic jar. Then, the cultures were harvested by centrifugation (8000 ×* g*, 10 min), washed twice with 0.9% saline, and adjusted to an optical density at 600 nm (OD_600_) of 0.7 in saline. One milliliter of solvent (xylene or toluene) was added to each bacterial suspension, and the mixtures were vortexed for 2 min and then incubated for 2 h at 37°C. The lower aqueous phase was carefully removed, and the A_600_ was read in a spectrophotometer. Hydrophobicity was calculated using the following formula: hydrophobicity (%) = ((A_0_-A_2_)/A_0_) × 100, where A_0_ is the absorbance at time 0 (0 h) and A_2_ is the absorbance after 2 h.

### 2.5. *Lactobacillus* Adhesion to HMVII Cells

To verify the adhesion capacity of the* Lactobacillus *strains to HMVII cells, the method of Santos et al. [16] was used, with some modifications. Initially, a monolayer of HMVII cells at a concentration of 1 × 10^6^ cells/well was grown in 24-well plates in an incubator (SANYO) at 37°C and 5% CO_2_, and the lactobacilli were grown in MRS broth for 18 h at 37°C in anaerobic jar. After culture, the lactobacilli were recovered by centrifugation (8000 ×*g*, 10 min), washed twice with 0.9% saline solution (w/v), and adjusted to 10^8^ CFU/mL in RPMI supplemented with 10% fetal bovine serum (FBS).* Lactobacillus* suspensions were added to wells containing HMVII cells and were incubated for 2 h at 37°C and 5% CO_2_. Subsequently, the cells were washed three times with 0.9% saline solution (w/v) and removed from the plates with 0.25% trypsin-EDTA for 5 min. The percentage of adhered lactobacilli was determined by plating serial dilutions on MRS agar. The plates were incubated for 48 h at 37°C, and then the bacteria (CFU/mL) were counted. The percentage of adherent lactobacilli was calculated by the following formula: adhesion (%) = (final CFU_end_/CFU_initial_) × 100.

To visualize the adhesion of the* Lactobacillus* strains to HMVII cells, scanning electron microscopy (SEM) was performed. HMVII cells (1 × 10^6^ cells/well) were grown on 24-well plates (containing glass coverslips in each well) in an incubator (SANYO) at 37°C and 5% CO_2_, with one of the two tested* Lactobacillus *strains (1 × 10^8^ CFU/mL) and incubated for 2 h at 37°C and 5% CO_2_. HMVII cells alone were used as controls. After incubation, the coverslips were washed three times with 0.9% saline solution to remove the nonadherent lactobacilli cells.

### 2.6. Evaluation of Acid Production by Lactobacilli

To evaluate acid production by lactobacilli the method of Pessoa el al. was used [18]. Culture supernatants of the* Lactobacillus *strains were obtained to evaluate acid production. The cultures were grown in MRS broth for 48 h at 37°C and then centrifuged (8000 ×* g*, 15 min). The supernatant was separated from the pellet, and the pH was measured with a pH meter.

### 2.7. Susceptibility of Lactobacilli to Antibiotics

The antibiotics susceptibility profiles of the* Lactobacillus *strains were determined by using the modified agar diffusion method of Clinical and Laboratory Standards Institute (CLSI) [22]. Lactobacilli were grown in MRS broth for 18 h at 37°C in anaerobic jar and then centrifuged (8000 ×* g*, 10 min). The cell pellets were washed twice with 0.9% saline solution (w/v) and adjusted to 0.5 MacFarland. Then, 100 *μ*L of this suspension was spread on MRS agar plates, and antibiotic disks were placed on the plates. The plates were incubated at 37°C for 24 h, and then the diameter of the zone of inhibition surrounding each disk was measured and classified as sensitive (S), moderately sensitive (MS), or resistant (R), according to Charteris et al. [23]. The antibiotics tested were amoxicillin (10 *μ*g), ampicillin (10 *μ*g), cephalothin (30 *μ*g), ciprofloxacin (5 *μ*g), clindamycin (2 *μ*g), chloramphenicol (30 *μ*g), erythromycin (10 *μ*g), gentamicin (10 *μ*g), nitrofurantoin (300 *μ*g), norfloxacin (10 *μ*g), penicillin G (10 *μ*g), tetracycline (30 *μ*g), and vancomycin (30 *μ*g).

### 2.8. Interactions between HMVII Cells and LAMP and the Effect of* Lactobacillus* Strains

To assess the interactions between* Mycoplasma* and* Ureaplasma *LAMP and HMVII cells, first a monolayer of HMVII cells, at a concentration of 1 × 10^6^ cells/well, was grown in 24-well plates in an incubator (SANYO) at 37°C and 5% CO_2_. After 24 h, the interactions between the lactobacilli strains and the lipoproteins tested were added to the wells of the plates, according to Table 1. The concentration of LAMP used in study (4 *μ*g/mL) was determined from previous tests evaluating the dose response of HMVII cells to the lipoproteins (data not shown). And the suspensions of lactobacilli were adjusted to 1 × 10^8^ CFU/mL in RPMI supplemented with 10% FBS.

### 2.9. Flow Cytometry to Assess the Viability of HMVII Cells

After the HMVII cells were incubated with the* Lactobacillus *strains and/or lipoproteins for 24 h, they were disrupted by trypsinization, washed twice with PBS and collected by centrifugation (600 x* g*, 10 min). The cells were labelled using LIVE/DEAD™ Viability/Cytotoxicity Kit for mammalian cells (Invitrogen/Thermo Fisher Scientific, Carlsbad, CA, USA) according to the manufacturer's instructions. Fluorescence was analyzed on a FC 500 flow cytometer (Beckman Coulter) with 20,000 events.

### 2.10. Scanning Electron Microscopy (SEM)

Scanning electron microscopy was performed to confirm* Lactobacillus* adhesion to the vaginal cells (after 2 h), as well as demonstrate the physical integrity of the HMIVII cells after interaction with the lipoproteins and* Lactobacillus *strains (after 24 h). After the incubation period, the culture supernatant was removed from each well, and the cells on the coverslips were fixed with 2.5% glutaraldehyde in 0.1M cacodylate buffer for at least 4 h. Next, the samples were washed with 0.1M cacodylate buffer for 5 min, twice, and then dehydrated with increasing concentrations of acetone (50–100%) for 10 min. After dehydration, the samples were taken to the critical point chamber and mounted on the sample holder of the “Stub” SEM with double carbon tape. Then, they were sputter coated with a thin layer of gold, about 20–30 nm thick, with a Sputter Coater SCD 050 (Baltec) for observation in a Quanta 250 Scanning Electron Microscope (FEI Company).

### 2.11. Statistical Analysis

All analyses were performed in triplicate. Quantitative data are expressed as the mean and standard deviation and analyzed with GraphPad Prism software (version 5.01).

For the hydrophobicity, autoaggregation, and adhesion tests, the statistical differences were determined by t-test followed by the Mann-Whitney posttest, and a p value less than 0.05 was considered significant. The statistical differences in the flow cytometry results were determined by ANOVA followed by Tukey's posttest, and a p value less than 0.01 was considered significant.

## 3. Results and Discussion

### 3.1. Evaluation of the Probiotic Properties of Lactobacilli Isolated from Cocoa Fermentation

To test whether the test* Lactobacillus *strains had probiotic properties, their surface properties, including autoaggregation and hydrophobicity, adherence to vaginal epithelial cells, and acid production in culture, were evaluated.

Autoaggregation percentages for* L. fermentum* FA4 and* L. plantarum* PA3 were 28.55% and 31.82%, respectively (Table 2). Bacterial autoaggregation, defined as the ability of cells to form precipitates, is considered an important probiotic property, as it is directly related to adhesion to host cell surfaces, one of the mechanisms by which probiotics compete with pathogenic bacteria [24]. These values were within the expected range, since there is great variation in autoaggregation among strains of both human vaginal microbiota and nonhuman origin [25, 26]. Gómez et al. [27] found autoaggregation values ranging from 20 to 70%, after only 24 h of incubation, among 8 strains of LAB isolated from different food sources, and the highest percentage, 67%, was a* Weissella viridescens *strain isolated from mature cheese. Similar to our study, the two strains of* L. plantarum* and one strain of* L. fermentum* isolated from cocoa fermentation showed autoaggregation values of approximately 29%, 33%, and 31% after 5 h of incubation [18].

The hydrophobicity or microbial adhesion to hydrocarbons (MATH) can be classified as low (MATH <33%), medium (33% <MATH <66%), or high (MATH> 66%) [28]. The hydrophobicity of* L. fermentum* FA4 was considered to be average for both xylene (57.03%) and toluene (65.33%), while that of* L. plantarum* PA3 was considered high for both xylene and toluene (66.75 and 71.12%, resp.). Some authors present hydrophobicity as microbial adhesion to solvents (MATS), classifying the bacterial surface as hydrophobic (MATS ≥55.00%), amphiphilic (45.00% ≤MATS≤54.99%), or hydrophilic (MATS ≤44.99%) [29, 30]. According to this classification,* L. fermentum* FA4 and* L. plantarum *are considered to be hydrophobic. This methodology is a simple way of evaluating the hydrophobicity of potential probiotic cell lines, indicating their ability to adhere to apolar surfaces, such as epithelial cell membranes. Their hydrophobicity justifies the application of these bacteria in probiotic formulations [30]. Similar results were obtained in other studies of isolates from nonhuman sources. For example, Cui et al. [31] determined hydrophobicities of 44% to 78% in lactobacilli isolated from artisanal cheese. In the present study, lactobacilli isolated from a cocoa fermentation showed higher hydrophobicities than several isolates of intestinal origin. In a study conduced by Yadav et al. [32], the highest hydrophobicity among* L. plantarum* isolates from human feces was 39.49%.

**Figure 1:**
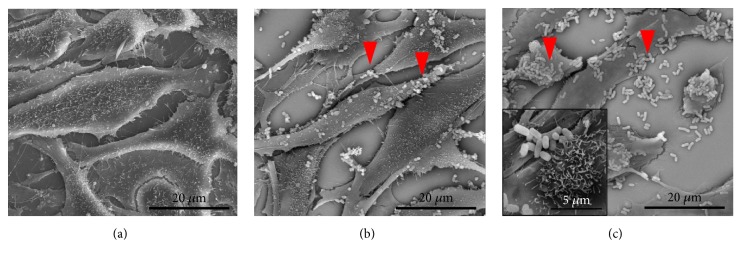
**Adhesion of lactobacilli isolated from cocoa fermentation to vaginal epithelial cells after 2 h of incubation.** (a) Untreated HMVII cells (×5000); (b) HMVII cells incubated with* L. fermentum* FA4 (×5000); (c) HMVII cells incubated with* L. plantarum* PA3 (×5,000, details at ×20,000). Red arrows indicate the lactobacilli attached to the cell surface.

The percentage of cells that adhered to the vaginal cells was 31.66% for* L. fermentum* FA4 and 41.65% for* L. plantarum* PA3 after 2 h of incubation. Adhesion was confirmed by SEM, in which it was possible to observe lactobacilli adhered to the surface of the HMVII cells (Figure 1). Adhesion is considered to be one of the major properties of a probiotic strain, because it is thought that the longer the strain remains adhered to the surface of the host cells, the more benefits it can confer. Probiotics are able to induce the expression of adhesins, such as mucin, fibronectin, collagen, laminin, and fibrinogen, which mediate adhesion [32, 33]. Strains of* Lactobacillus paracasei* subsp.* paracasei* produce an aggregation promoting factor (AggLb) that contributes to its high aggregation capacity, as well as a strong, specific interaction with host cell collagen, indicating that there is a direct relationship between cell aggregation, hydrophobicity, and collagen binding, as was observed in the* Lactobacillus *strains used in this study [34].

By evaluating the pH of the culture supernatants, we observed that the two* Lactobacillus* strains were able to reduce the pH of the culture medium from an initial pH=6.6 to similar pH values of 4.32 for* L. fermentum* FA4 and 4.33 for* L. plantarum* PA3 (Table 2). This reduction in pH by lactobacilli is mediated by the production of acids, mainly lactic and acetic acids, and is one of the mechanisms by which the growth of pathogenic bacteria is inhibited [35, 36]. In Gram-negative bacteria, lactic acid acts as a permeator of the bacterial outer membrane, releasing lipopolysaccharides (LPS), and increasing their susceptibility to other antibiotics produced by the host [37]. According to the ability to ferment sugars lactobacilli can be classified into homofermentative species (e.g.,* L. plantarum*), which convert sugars into lactic acid, and heterofermentative species (e.g.,* L. fermentum*), which produce lactic and acetic acids, ethanol, and CO_2_ [38]. Thus, contrary to what was observed in the present study, other authors have reported variations in the pH values of the culture medium for different species, indicating differences in acid production and secretion profiles; generally,* L. plantarum* strains tend to produce more acid than* L. fermentum* strains [18]. Among vaginal* Lactobacillus *isolates with inhibitory potential against* G. vaginalis*, an* L. fermentum* strain reduced the pH of the culture supernatant to pH 4.16 after 48 h of incubation [39], whereas 2 strains of* L. plantarum* isolated from organic fertilizer, in the same conditions for 24 h, reduced the pH to 3.83 and 3.88 [40]. Two strains of* L. plantarum* isolated from a cocoa fermentation reduced the pH to 3.81 and 3.77, whereas an* L. fermentum *isolate reduced the pH to 4.78 [18].

The tested* L. fermentum* FA4 and* L. plantarum* PA3 strains did not show any significant differences in the four parameters evaluated, indicating that they have similar probiotic potential.

### 3.2. Susceptibility of Lactobacilli to Antibiotics

In the antibiotics sensitivity profile testing, the two* Lactobacillus *strains were sensitive or moderately sensitive to most of the antibiotics tested. They were only resistant to vancomycin, aminoglycosides, and quinolones (Table 3). Although lactobacilli are “generally recognized as safe”, safety tests, such as the determination of antibiotics sensitivity profiles, should always be performed to avoid the transfer of resistance genes, since these profiles vary among species [41]. Similar sensitivity profiles have been described in other studies, corroborating the findings of the present study. Santos et al. [42] found that* L. fermentum* and* L. plantarum* isolates from cocoa fermentation were resistant to vancomycin and quinolone class antibiotics. Similarly, 12 lactobacilli strains isolated from cottage cheese, typical of northeastern China, were resistant to streptomycin, gentamicin, vancomycin, and ciprofloxacin [31]. Strains of human origin also have similar sensitivity profiles. Bouridani et al. [25] showed that all tested vaginal* Lactobacillus *isolates were resistant to ofloxacin, gentamicin, and ciprofloxacin, and almost all strains were sensitive to trimethoprim-sulfamethoxazole, ampicillin, erythromycin, cefotaxime, chloramphenicol, tetracycline, and nitrofurantoin. Bacteria in the genus* Lactobacillus*, like other Gram-positive bacteria, are intrinsically resistant to glycopeptides such as vancomycin. However, the gene responsible for this resistance is chromosomal, and it is not inducible or transferable to other bacteria [43, 44]. Thus, the strains under study could be used in therapeutic applications, as they would not pose a risk to the health of humans or other animals.

### 3.3. Interaction of HMVII Cells with Lipoproteins and the Effects of Treatment with Lactobacilli

**Figure 2:**
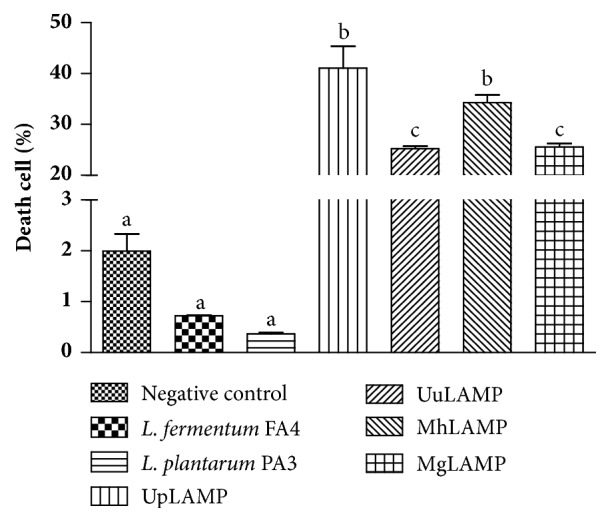
**Death of HMVII cells after 24 h of incubation with* Lactobacillus *strains or membrane-associated lipoproteins (LAMP) from* U. parvum* (UpLAMP),* U. urealyticum* (UuLAMP),* M. hominis* (MhLAMP), or* M. genitalium* (MgLAMP).** Different letters indicate significant differences (p <0.01).

Initially, we evaluated whether lactobacilli isolated from cocoa fermentation reduced the viability of cells of vaginal lineage. After 24 h of incubation with* L. fermentum* FA4 and* L. plantarum* PA3, the HMVII cells showed cell death rates of only 0.72% and 0.36%, respectively, and these rates were not significantly different from each other or from that of the control (1.99%; Figure 2).

This minimal reduction in HMVII cell viability after incubation with these* Lactobacillus* strains demonstrates that both can exist in the human vaginal environment without any toxicity. In addition, they reinforce the probiotic potential of these strains in protecting against pathogens that cause BV. Similarly, Abramov et al. [45] showed that* Lactobacillus crispatus* 2029 did not induce apoptosis in vaginal epithelium cells (VK2/E6E7) and has no cytotoxic effects on them. Strains of* L. fermentum* and* L. plantarum *inhibited apoptosis in HeLa epithelial cells and showed antagonistic activity against* Candida albicans* and* G. vaginalis* [46]. In another study, the viability of cells isolated from epidermoid carcinoma of the cervix (CaSki) and HeLa cells was maintained after incubation with different concentrations of* Lactobacillus casei* extract [47]. In contrast, cell death of HMVII cells increased significantly after incubation with the membrane-associated lipoproteins (LAMP) of* U. parvum*,* U. urealyticum*,* M. hominis*, and* M. genitalium*; LAMP extracted from* U. parvum *and* M. hominis *induced 41% and 34% cell death, respectively, the highest values among the 4 species (Figure 2).

*Mollicutes* causing genital infections need to adhere and subsequently invade the cells of the genitourinary tract to obtain nutrients from the host cells [1, 2, 47]. During adhesion, human cells first interact with the plasma membrane of these microorganisms, specifically with their lipoproteins. Recently, cytoadhesion and invasion present on the membrane of* M. hominis *were shown to interact with HeLa cells [48]. Hopfe et al. [49] showed that, after 4 h of infection of HeLa cells with* M. hominis*, genes related to the cell cycle, growth, and cell death are highly regulated, and lipoproteins are generally responsible for inducing inflammation, apoptosis, and cell death [5, 50].

The present study is the first to use the HMVII vaginal cell line as a model for interaction with the lipoproteins of* Mollicutes*, mimicking the infection that occurs* in vivo*. However, the levels of cell death induced by lipoproteins extracted from mycoplasmas and ureaplasmas may vary according to both species and the human cell line used in the experiment. Similarly, to the present study, 2 *μ*g/mL* Mycoplasma penetrans* lipoproteins induced apoptosis in RAW264.7 murine macrophages [51]. LAMP from* Mycoplasma salivarium* and* Mycoplasma fermentans* caused both apoptosis and necrosis in myeloid (HL-60 and THP-1) and lymphoid (MOLT-4 and Raji cells) leukemia cells at concentrations of 20 and 7 *μ*g/mL, respectively [52]. However, Bai et al. (2007) [4] showed that* Mycoplasma hyopneumoniae *LAMP induced apoptosis in the 3D4/21 porcine alveolar macrophage (PAM) cell line at concentrations of 0.4 mg/mL or higher, which is higher than the concentration used in the present study.

**Figure 3:**
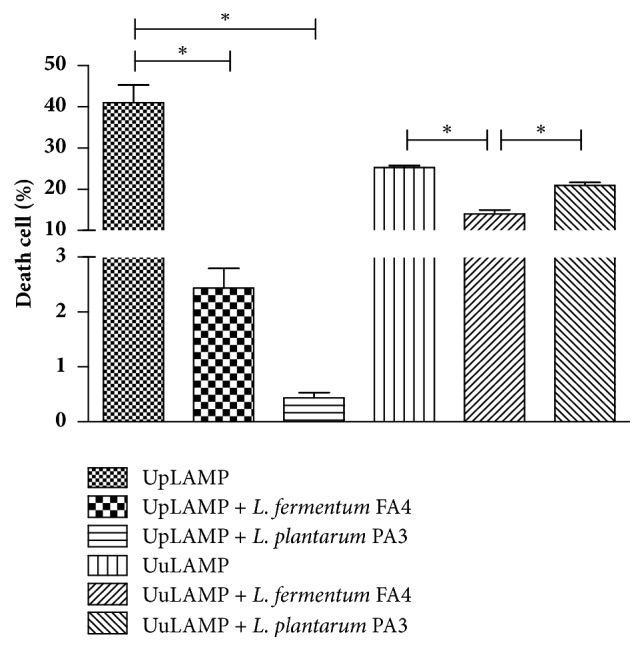
**Death of HMVII cells after 24 h of incubation with LAMP from* U. parvum* (UpLAMP) or* U. urealyticum* (UuLAMP) with and without* L. plantarum* PA3 or* L. fermentum* FA4.** *∗p*<0.01 compared to cells with LAMP alone.

**Figure 4:**
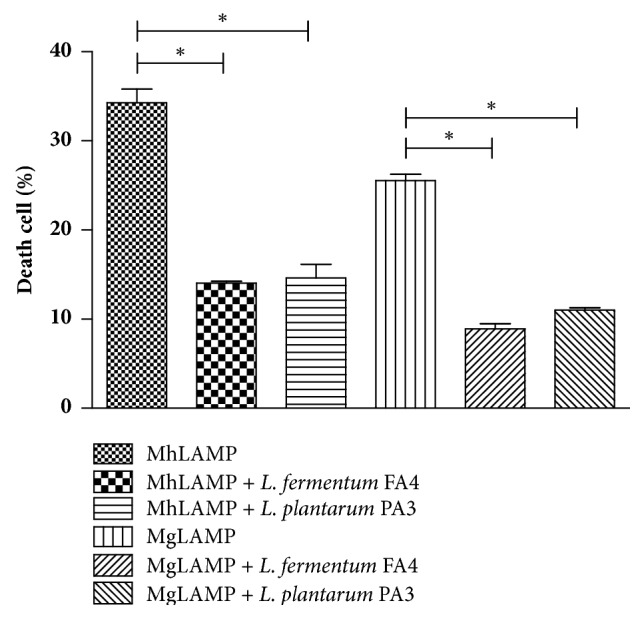
**Death of HMVII cells after 24 h of incubation with LAMP from* M. hominis* (MhLAMP) or* M. genitalium *(MgLAMP) with and without* L. plantarum* PA3 or* L. fermentum* FA4.** *∗p*<0.01 compared to cells with LAMP alone.

In this study, we also demonstrated that incubation of HMVII cells with lactobacilli isolated from cocoa fermentation significantly reduced cell death caused by the interaction with* Ureaplasma* species LAMP from 41.03% in UpLAMP to 2.43% (UpLAMP +* L. fermentum* FA4) and 0.43% (UpLAMP +* L. plantarum* PA3) and from 25.24% (UuLAMP) to 13.97% (UuLAMP +* L. fermentum* FA4) (Figure 3). As described for the genus* Ureaplasma*, the treatment of HMVII with lactobacilli also managed to significantly decrease the rate of cell death caused by the interaction with LAMP of the genus* Mycoplasma*. The cell death reduced from 34.29% (MhLAMP) to 14.06% (MhLAMP +* L. fermentum* FA4) and 14.61% (MhLAMP +* L. plantarum* PA3) and 25.53 (MgLAMP) to 8.91% (MgLAMP +* L. fermentum* FA4) and 11% (MgLAMP +* L. plantarum* PA3) (Figure 4).

**Figure 5:**
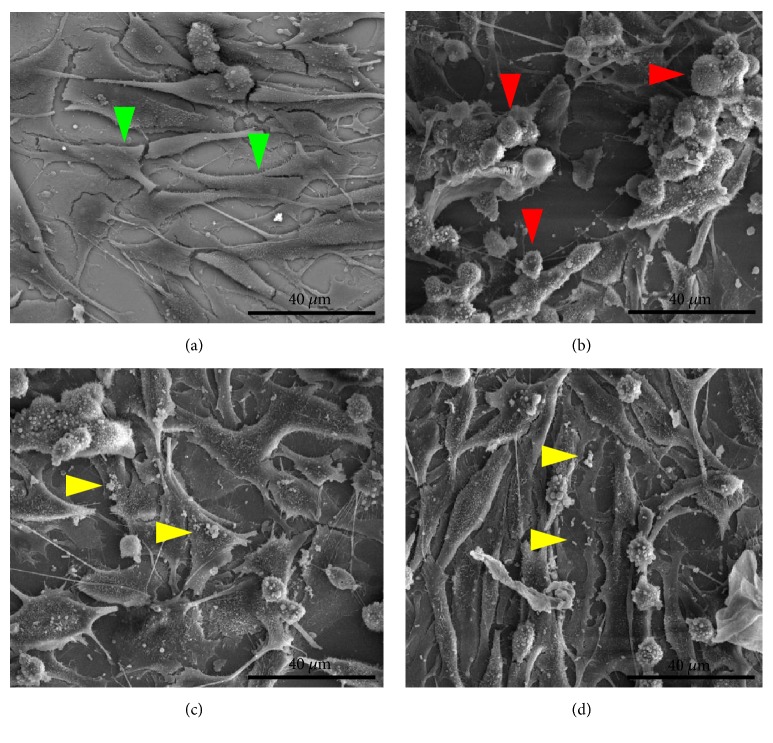
**Interaction of HMVII cells with 4 **μ**g/mL of* U. parvum *LAMP with and without* L. fermentum* FA4 or* L. plantarum* PA3. **(a) Control (HMVII cells alone). (b) HMVII with UpLAMP. (c) HMVII with UpLAMP and* L. fermentum* FA4. (d) HMVII with UpLAMP and* L. plantarum* PA3. Green arrows indicate intact HMVII cells, red arrows indicate HMVII cells with altered morphology, and yellow arrows indicate lactobacilli adhered to whole cells (scanning electron microscopy, ×2500).

**Figure 6:**
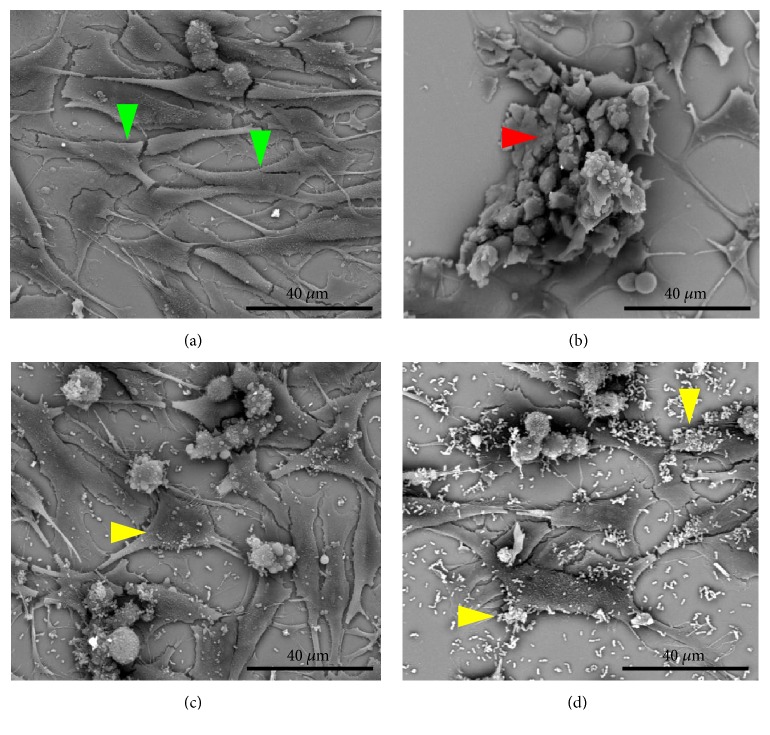
**Interaction of HMVII cells with* U. urealyticum *LAMP with and without* L. fermentum* FA4 or* L. plantarum* PA3. **(a) Control (HMVII cells alone). (b) HMVII with UuLAMP. (c) HMVII with UuLAMP and* L. fermentum* FA4. (d) HMVII with UuLAMP and* L. plantarum* PA3. Green arrows indicate intact HMVII cells, red arrows indicate HMVII cells with altered morphology, and yellow arrows indicate lactobacilli adhered to whole cells (scanning electron microscopy, ×2500).

**Figure 7:**
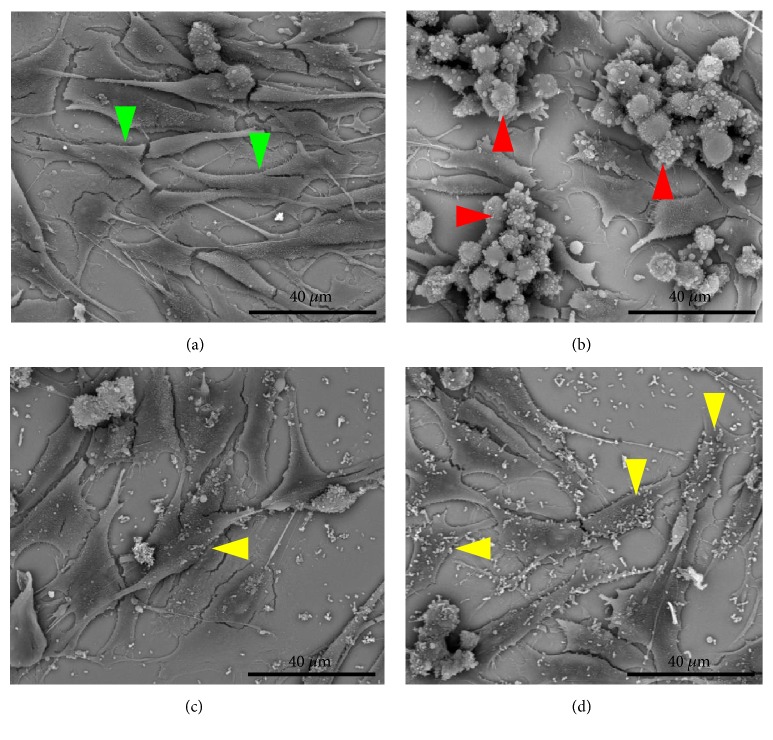
**Interaction of HMVII cells with 4 **μ**g/mL of* M. hominis *LAMP with and without* L. fermentum* FA4 or* L. plantarum* PA3.** (a) Control (HMVII cells alone). (b) HMVII with MhLAMP. (c) HMVII with MhLAMP and* L. fermentum* FA4. (d) HMVII with MhLAMP and* L. plantarum* PA3. Green arrows indicate intact HMVII cells, red arrows indicate HMVII cells with altered morphology, and yellow arrows indicate lactobacilli adhered to whole cells (scanning electron microscopy, ×2500).

**Figure 8:**
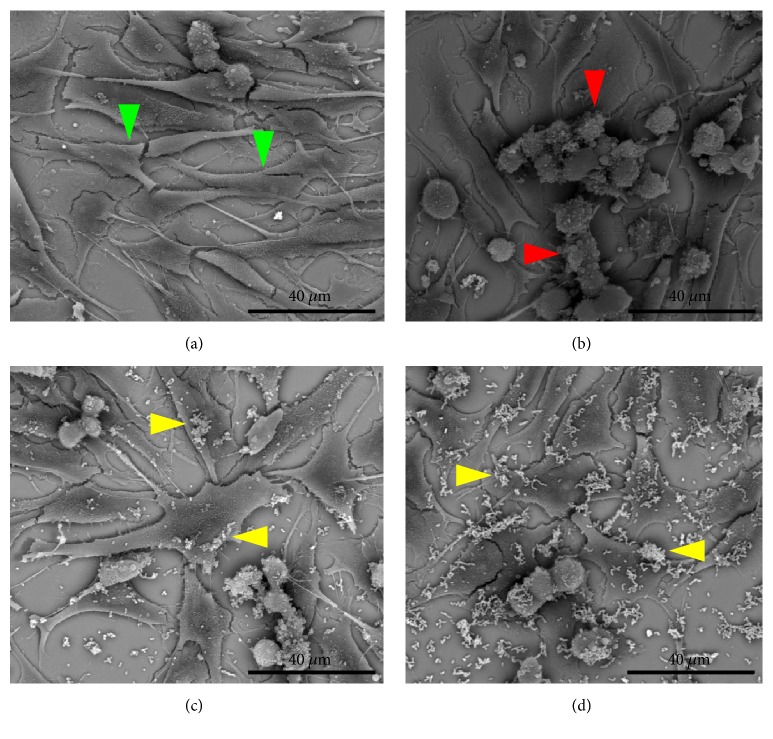
**Interaction of HMVII cells with 4 **μ**g/mL* M. genitalium* lipoproteins with and without* L. fermentum* FA4 or* L. plantarum* PA3.** (a) Control (HMVII cell alone). (b) HMVII with MgLAMP. (c) HMVII with MgLAMP and* L. fermentum* FA4. (d) HMVII with MgLAMP and* L. plantarum* PA3. Green arrows indicate intact HMVII cells, red arrows indicate HMVII cells with altered morphology, and yellow arrows indicate lactobacilli adhered to whole cells (scanning electron microscopy ×2500).

The findings of flow cytometry were confirmed by microscopy (Figures 5–8). The images showed the vaginal cells after interaction with lipoproteins extracted from* U. parvum*,* U. urealyticum*,* M. hominis,* and* M. genitalium* (resp.) and treatment with the two strains of lactobacilli. Cells incubated only with lipoproteins at a concentration of 4 *μ*g / mL (Figures 5(b)–8(b)) presented a large number of cells with altered morphology, when compared to the control (Figures 5(a)–8(a)).

In contrast, in treatments with L. fermentum FA4 (Figures 5(c)–8(c)) and L. plantarum PA3 (Figures 5(d)–8(d)), bacteria adhered to HMVII cells were able to reduce cell death. This property was evidenced by normal morphological characteristics of the cells compared to the negative control.

Several studies have shown that lactobacilli isolated from the vaginal environment have activity against potentially pathogenic bacteria. Strains of* L. crispatus* and* L. vaginalis* inhibited the growth of several bacterial species causing vaginal and urinary tract infections, including* Enterococcus faecalis*,* E. coli*,* S. aureus*,* Enterococcus faecium*,* G. vaginalis*, and* Proteus mirabilis *[53]. In addition to promoting a protective vaginal environment, strains of* L. crispatus *showed antagonistic activity against several species of the genus* Candida* [45]. Strains of* Lactobacillus rhamnosus* and* Lactobacillus reuteri* increased the viability of human epidermal keratinocytes from 8.8% after infection with* S. aureus* to 42.7% and 53.1%, respectively [54]. However, very few studies have examined the interaction between lactobacilli and* Mollicutes*. Danielle et al. [55] demonstrated that bacteriocins produced by* L. fermentum* and* L. rhamnosus* showed antibacterial activity against vaginal isolates of* U. urealyticum* and* M. hominis*. This is the first study to investigate the interaction between* Lactobacillus *strains isolated from cocoa fermentation and the* Mollicutes *class of bacteria. The antagonist effects of probiotic microorganisms against pathogenic bacteria include competitive adhesion to the mucosa and epithelium; strengthening of the epithelial barrier; secretion of antibiotics substances, such as bacteriocins and organic acids; and modulation of the immune system [36].

One possible protective mechanism of the strains in this study against* Mycoplasma* and* Ureaplasma* lipoproteins is competitive adhesion to host epithelial cells. The excellent adhesion capacity and hydrophobicity of the strains in this study suggest antagonistic effects on the adhesion of LAMP to the plasma membrane of the vaginal epithelium. In addition, this high hydrophobicity, coupled with the self-aggregation ability, of the tested lactobacilli may cause the LAMP to bind to the cell wall of these microorganisms rather than to the epithelial cell membrane [35]. Finally, the cell interaction experiment results indicate that these* L. fermentum* and* L. plantarum* strains isolated from cocoa fermentation have potential for use as vaginal cell protectors against the LAMP of several important pathogens of the genitourinary tract, including* U. parvum*,* U. urealyticum*,* M. hominis*, and* M. genitalium*. However, more studies are needed to clarify their protective mechanism(s) of action.

## 4. Conclusions

In the present study, lactobacilli isolated from cocoa fermentation inhibited cell death of a vaginal cell line (HMVII) induced by* Mollicutes* lipoproteins (*Mycoplasma* and* Ureaplasma* strains) that cause genital infections. The tested* Lactobacillus *strains have the fundamental and desirable probiotic properties required to maintain a healthy vaginal environment, including high hydrophobicity and autoaggregation, as well as adherence to epithelial cells and acid production. These characteristics are considered promising for the development of further prophylactic agents.

## Figures and Tables

**Table 1:** Interactions between vaginal cells and *Mollicutes *lipoproteins in the presence and/or absence of lactobacilli isolated from cocoa fermentation.

**Lipoproteins**	**Lactobacilli**	
	***Lactobacillus fermentum* FA4**	***Lactobacillus plantarum* PA3**
***Ureaplasma parvum*** **(UuLAMP)**	UpLAMP + *L. fermentum* FA4	UpLAMP + *L. plantarum* PA3
***Ureaplasma urealyticum*** **(UpLAMP)**	UuLAMP + *L. fermentum* FA4	UuLAMP + *L. plantarum* PA3
***Mycoplasma hominis*** **(MhLAMP)**	MhLAMP + *L. fermentum* FA4	MhLAMP + *L. plantarum* PA3
***Mycoplasma genitalium*** **(MgLAMP)**	MgLAMP + *L. fermentum* FA4	MgLAMP + *L. plantarum* PA3

**Table 2:** The surface properties of lactobacilli isolated from cocoa fermentation, their adhesion to vaginal cells, and acidification of the culture medium.

**Strain**	**Surface properties**			**Adhesion to HMVII cells (**%**)**	**pH value**
	**Autoaggregation (**%**)**	**Hydrophobicity (**%**)**			
		**Xylene**	**Toluene**		
*L. fermentum* FA4	28.55 ± 1.08	57.03 ± 1.80	65.33 ± 0.99	31.66 ± 7.82	4.32
*L. plantarum* PA3	31.82 ± 0.58	66.75 ± 5.11	71.12 ± 3.31	41.65 ± 13.85	4.33

**Table 3:** Antibiotics susceptibility profiles of *Lactobacillus* strains isolated from cocoa fermentation.

Antibiotics			Susceptibility^a^	
Group	Name	Antibiotic concentration (*μ*g)	*L. fermentum *FA4	*L. plantarum *PA3
*Inhibitors of cell wall synthesis*				

Penicillin	Amoxicillin	10	S	S
	Ampicillin	10	S	S
	Penicillin G	10	S	MS
Cephalosporins	Cefalotin	30	S	S
Glycopeptides	Vancomycin	30	R	R

*Inhibitors of protein synthesis*				

Aminoglycosides	Amikacin	30	R	R
	Gentamicin	10	R	R
	Streptomycin	10	R	R
Tetracyclines	Tetracycline	30	S	S
Single antibiotics	Chloramphenicol	30	S	S
Macrolides	Erythromycin	15	S	S
Lincosamides	Clindamycin	2	S	S

*Inhibitors of nucleic acid synthesis*				

Quinolones	Ciprofloxacin	5	R	R
	Norfloxacin	10	R	R

*The broad mechanism of action*				

Single antibiotics	Nitrofurantoin	300	S	S

## Data Availability

The data used to support the findings of this study are included within the article.
